# Heterogeneous network drug-target interaction prediction model based on graph wavelet transform and multi-level contrastive learning

**DOI:** 10.1038/s41598-025-16098-y

**Published:** 2025-08-19

**Authors:** Wenfeng Dai, Yanhong Wang, Shuai Yan, Qingzhi Yu, Xiang Cheng

**Affiliations:** School of Information Engineering, Jingdezhen Ceramics University, Jingdezhen, Jiangxi 333403 China

**Keywords:** Heterogeneous networks, Graph wavelet transform, Heterogeneous graph convolutional network, Contrastive learning, Attention mechanism, Computational biology and bioinformatics, Drug discovery

## Abstract

Reliable prediction of drug–target interaction (DTI) is essential for accelerating drug discovery, yet remains hindered by data imbalance, limited interpretability, and neglect of protein dynamics. Here, we present **GHCDTI**, a heterogeneous graph neural framework designed to overcome these challenges through three synergistic innovations. First, **cross-view contrastive learning** with adaptive positive sampling improves generalization under extreme class imbalance (positive/negative ratio<1:100). Second, **heterogeneous data fusion** integrates molecular graphs, protein structure graphs, and bioactivity data via cross-graph attention, enabling interpretable residue-level insights. Third, **multi-scale wavelet feature extraction** captures both conserved and dynamic structural features by decomposing protein conformations into frequency components. GHCDTI achieves state-of-the-art performance on benchmark datasets (AUC: 0.966 ± 0.016; AUPR: 0.888 ± 0.018) and processes 1,512 proteins and 708 drugs in under two minutes, highlighting its potential for scalable virtual screening and drug repositioning. These results demonstrate GHCDTI’s ability to effectively identify novel drug–target pairs, providing a practical tool for accelerating drug discovery and improving biomedical knowledge integration.

## Introduction

Drugs, as small-molecule agents that modulate target protein functions, serve as the cornerstone of modern disease treatment. However, drug development requires 10-15 years and costs approximately US$2.6 billion on average. Within this process, the prediction of DTI critically determines the efficiency of the screening of lead compounds^1^. Computer-aided drug design (CADD)^2^ has revolutionized traditional approaches by establishing structure-activity relationship models that integrate multi-dimensional biomarkers.

Within the CADD framework, DTI prediction methods have achieved significant breakthroughs, evolving along three main technical pathways: (1) Structure-based computational approaches–the classical strategy–employ molecular docking and free energy calculations to simulate drug–target geometric conformations, though constrained by high computational costs^3–5^. For instance, Zhang et al.^6^ used structure-based virtual screening methods to identify potential highly active compounds, thereby accelerating the process of new drug design. Notably, Su et al.^7^ calculated the absolute binding free energy by integrating physical laws and geometric knowledge to establish a robust protein-ligand interaction model. (2) Phenotype-based deep learning methods–emerging as a computationally efficient alternative–leverage graph neural networks (GNNs) and Transformer architectures to decode nonlinear patterns directly from compound activity profiles^8–10^. Complementing structure-based methods, Zhang et al.^11^ presented a transformer-based approach that incorporates multilayer graph information for DTI prediction. Huang et al.^12^ further advanced this paradigm by developing an enhanced Transformer encoder that distills semantic relationships from unlabeled biomedical substructures. Notably, Abbasi et al. used generative adversarial networks to design optimized drug candidates^13^. (3) Hybrid intelligent systems–bridging data-driven and physics-based paradigms–strategically combine quantitative structure-activity relationship (QSAR) modeling with deep learning architectures. Building upon phenotype-based approaches, these methods achieve enhanced interpretability while maintaining computational efficiency. The SwissADME platform exemplifies this synergy, integrating ADMET property predictors (e.g., absorption/distribution coefficients) with neural networks to deliver real-time bioavailability predictions^14–17^. Wu et al.^18^ proposed a multimodal attention-based DTA prediction model, AttentionMGT-DTA, which uses molecular graphs and binding pocket graphs to represent drugs and targets, respectively, and adopts two attention mechanisms to integrate and interact information between different protein modalities and drug-target pairs. Building further upon recent multimodal learning advances, Yang et al.^19^ introduced Modality-DTA, a novel framework that integrates multiple modalities of drug and target data to capture complementary information.

Despite the significant progress of CADD technology, deep learning-driven DTI prediction still faces three challenges: data bias problem: the ratio of positive and negative samples in the DTI dataset is seriously unbalanced (usually $<1:100$), which leads to overfitting of the model in unseen compounds (ROC curve deviates significantly in the low activity region); interpretability dilemma: the black box model cannot quantify the contribution of key residues to binding energy; lack of dynamic characteristics: existing models mostly use static protein structures and fail to capture the impact of dynamic changes in target conformation on binding strength.

To address the critical limitations in current DTI prediction, namely, data imbalance, limited interpretability, and the absence of conformational dynamics modeling, we propose GHCDTI, a heterogeneous graph neural network framework that introduces three synergistic innovations:

1. **Multi-scale wavelet feature extraction**: We design a graph wavelet transform (GWT) module to decompose protein structure graphs into frequency components. Low-frequency filters capture the conserved global patterns associated with protein domains, while high-frequency filters highlight localized variations relevant to dynamic binding sites. This enables the model to effectively represent both structural stability and conformational flexibility.
2. **Heterogeneous data fusion**: GHCDTI constructs a unified heterogeneous graph that integrates molecular graphs of compounds (nodes: atoms; edges: chemical bonds), residue-level protein structure graphs (nodes: amino acids; edges: spatial distances) and external bioactivity data. A cross-graph attention mechanism is employed to align multi-source information across modalities. Additionally, semantic attention enhances dynamic context perception, resulting in improved interpretability and more accurate identification of key interaction regions.
3. **Cross-view contrastive learning**: To ensure robust representation learning under extreme class imbalance, we introduce a three-stage contrastive learning framework. Node-level representations are independently generated from a topological view (via a heterogeneous graph convolutional network) and a frequency-domain view (via GWT). These are aligned using InfoNCE loss to maximize agreement between corresponding nodes across views, promoting feature consistency and improving generalization on novel samples.

## Methods

### Dataset

The dataset from Luo et al.^20^ was used to construct a comprehensive heterogeneous biomedical network. This network includes four types of nodes and eight types of biologically meaningful edges, as summarized in Table 1.To maintain consistency across node and edge types, self-loop edges were added for each node to preserve identity information. Moreover, drug–drug similarity scores and protein sequence similarities were filtered by thresholding to eliminate weak connections and retain only biologically significant links. The resulting heterogeneous network provides a multi-scale relational foundation for downstream drug–target interaction prediction.

**Table 1:** Summary of Luo et al.’s heterogeneous biomedical network.

	Count
**Node Types**	
Proteins	1,512
Drugs	708
Diseases	5,603
Side effects	4,192
**Edge Types**	
Drug–drug interaction	5,018
Drug–side effect association	80,164
Drug–disease association	199,214
Drug–protein interaction	1,923
Protein–protein interaction	3,977
Protein–disease association	1,596,745
Drug–drug similarity	/
Protein–protein similarity	/

The dataset from Zeng et al.^21^ was also used to build another heterogeneous biomedical network. Table 2 summarizes the key statistics of this dataset. It integrates diverse biomedical relationships curated from multiple authoritative sources, including DrugBank, TTD, PharmGKB, ChEMBL, BindingDB, and IUPHAR/BPS.

**Table 2:** Summary of Zeng et al.’s heterogeneous biomedical network.

Relation Type	Entities Involved	Count
Drug–target interaction	732 drugs, 1,176 proteins	5,680
Protein–protein interaction	1,915 proteins	16,133
Drug–drug interaction	732 drugs	132,768

These heterogeneous relationships provide a robust foundation for downstream DTI prediction and systematic drug repurposing.

### Node feature construction

**Fig. 1:**
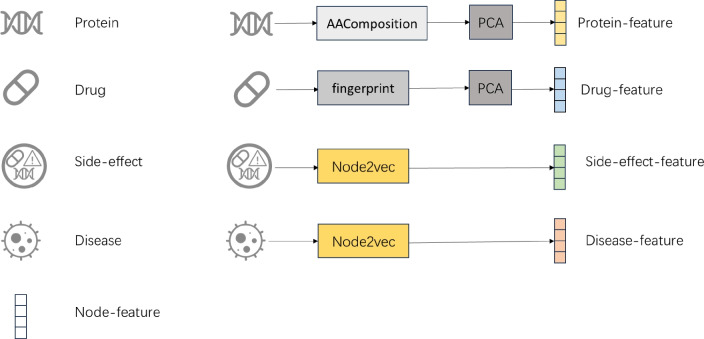
For drug nodes, SMILES molecular structures are extracted and converted into MACCS molecular fingerprints, followed by dimensionality reduction using principal component analysis (PCA)^22^ to obtain low-dimensional vector representations. For protein nodes, amino acid compositions and dipeptide frequencies are computed from their sequences and similarly reduced via PCA. For side effect and disease nodes, which lack inherent structural or sequential information, the Node2vec algorithm^23^ is adopted to generate embeddings. Specifically, a heterogeneous biomedical network is constructed based on known interactions, including drug–drug interactions, drug–disease associations, drug–side effect associations, protein–protein interactions, and protein–disease associations. Node2vec is applied to this network to capture the contextual semantics of side effect and disease entities through biased random walks.

We used the dataset from Luo et al.^20^ to build a heterogeneous biomedical network (Fig. 1) and constructed node features via molecular fingerprints, sequence-based statistics, and network embeddings. All node types are ultimately encoded as 128-dimensional vectors to ensure consistency for downstream prediction tasks.

### Model architecture

**Fig. 2:**
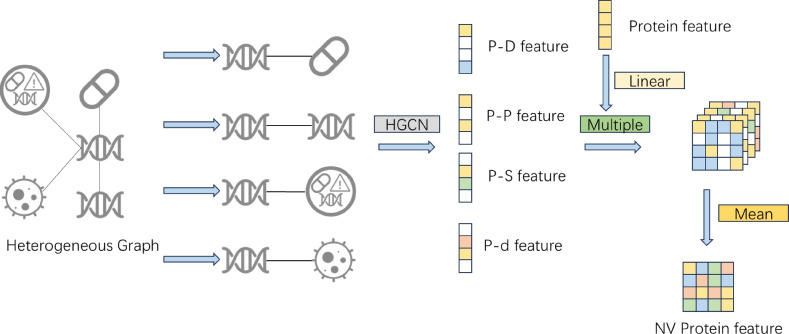
When extracting protein node features from the constructed heterogeneous network, the protein interactions are first divided into four edge types according to connected node categories: protein–drug, protein–protein, protein–side effect, and protein–disease. Subsequently, an HGCN is applied independently to each edge type to extract relational features. These learned features, together with the original protein features reduced via PCA, are integrated using a multi-modal fusion module, referred to as Multiple, which performs element-wise multiplication to combine modalities. Finally, the four fused features are aggregated through mean pooling to yield the final protein feature matrix. Drug features are processed in the same manner.

**Fig. 3:**
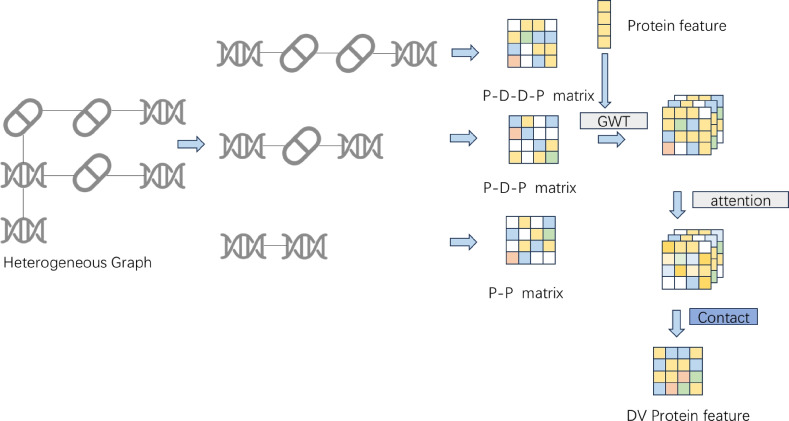
To comprehensively extract deep-level protein node features, this study employs a heterogeneous network to mine three key protein multi-hop pathways: direct protein–protein interactions (P–P), first-order indirect protein–drug–protein associations (P–D–P), and second-order mediated protein–drug–drug–protein relationships (P–D–D–P). First, we construct adjacency matrices for the three pathways separately and feed them, together with the PCA-reduced protein features, into the GWT module for multi-scale feature extraction. This enables simultaneous capture of both local and global topological information and yields multi-view protein feature representations. During the feature fusion stage, an attention mechanism is introduced to adaptively assign weights to features from the three pathways, followed by element-wise scalar product fusion (i.e., inner product of tensor vectors) to generate the final unified protein feature matrix. Drug features are processed in the same manner.

**Fig. 4:**
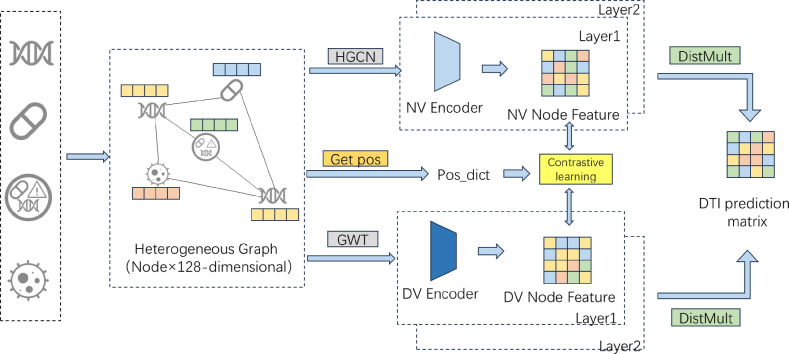
The proposed model begins by constructing a heterogeneous graph that integrates four types of biomedical entities–drugs, proteins, diseases, and side effects–along with their corresponding interactions. Initial node features, as illustrated in Fig. 1, are extracted and then combined with the constructed heterogeneous graph structure to serve as input to two parallel encoding modules: a two-layer NV encoder and a two-layer DV encoder. These modules capture complementary semantic perspectives from the heterogeneous network and generate enriched node-level representations. To align and enhance the expressiveness of the representations from both views, a contrastive learning mechanism is employed. Specifically, a similarity-based sampling strategy (Get Pos) is used to construct a dictionary of positive and negative samples (Pos_dict), which guides the contrastive optimization process between the NV and DV encoder outputs. Finally, the resulting node representations from both encoders are fused and passed into a DistMult decoder. This decoder incorporates multiple types of biomedical relations (e.g., drug–drug, protein–protein, drug–disease, etc.) through relation-specific diagonal matrices to reconstruct the full set of heterogeneous interactions. In particular, it focuses on computing the DTI matrix, which constitutes the primary prediction task of the model.

To accurately predict protein–target interactions, we propose a multi-perspective heterogeneous graph convolutional architecture. First, we extracted features for various node types from the dataset and constructed a heterogeneous network. The model then performs graph convolutional learning from both local and global perspectives: the neighborhood perspective captures direct local structures (Fig. 2), while the deep perspective explores higher-order node relationships through cross-type multi-hop paths (Fig. 3). Subsequently, node representations from these two perspectives are aligned and fused via multi-level contrastive learning. Finally, the integrated features are utilized to predict the drug–protein interaction matrix, enabling precise prediction of protein–target interactions. The overall architecture of the model is illustrated in Fig. 4, which comprises four key components: neighborhood-view encoder, deep-view encoder, multi-level contrastive learning module, and drug–target prediction module.

The model takes as input a heterogeneous biomedical graph ${\mathcal {G}} = ({\mathcal {V}}, {\mathcal {E}})$, where ${\mathcal {V}}$ includes four types of nodes: drugs, proteins, diseases, and side effects, and ${\mathcal {E}}$ contains eight types of biologically meaningful edges (e.g., drug–target, drug–disease, protein–protein). Each node $v_i \in {\mathcal {V}}$ is associated with a 128-dimensional initial feature vector ${\textbf{x}}_i \in {\mathbb {R}}^{128}$, constructed from domain-specific representations. These features are propagated through the dual encoders to learn multi-view node embeddings.

The final output of the model is a drug–target interaction (DTI) prediction matrix $\hat{{\textbf{Y}}} \in {\mathbb {R}}^{N_d \times N_p}$, where $N_d$ and $N_p$ denote the number of drugs and proteins, respectively. Each entry $\hat{y}_{ij} \in [0, 1]$ represents the predicted probability of interaction between drug $d_i$ and protein $p_j$.

#### Neighborhood-View Encoding (NV Encoder)

We adopt the Heterogeneous Graph Convolutional Network (HGCN), originally proposed by Wang et al.^24^ and further developed in heterogeneous graph modeling by Zhang et al.^25^, to aggregate neighborhood information and extract interaction-specific relational features from the constructed heterogeneous graph. HGCN is a neural architecture designed to process graph-structured data through feature propagation between adjacent nodes, thereby capturing both topological structures and node-level dependencies.

In our heterogeneous network, node representations are iteratively updated by aggregating typed neighbor features using an HGCN framework, following the principles introduced in^24^. The aggregation process is formally defined as follows:

1 $$\begin{aligned} H_v^{i} = \frac{1}{|N(v)| + 1} \left( \sum _{u \in N(v)} \widetilde{D}_{v,u}^{-\frac{1}{2}} \widetilde{A}_{v,u} \widetilde{D}_{v,u}^{-\frac{1}{2}} H_u^{i} W_{v,u} + H_v \right) \end{aligned}$$

Here, *N*(*v*) denotes the set of neighboring node types for node type *v*. $A_{v,u}$ represents the adjacency matrix between node types *v* and *u*, while $\widetilde{A}_{v,u} = A_{v,u} + I$ corresponds to the augmented adjacency matrix with self-connections. $\widetilde{D}_{v,u}$ is the degree matrix of $\widetilde{A}_{v,u}$, used for normalization. $H_u$ and $H_v$ denote the feature representations of neighbor node type *u* and the inherent features of node type *v*, respectively. $W_{v,u}$ is a trainable weight matrix that linearly transforms the features of neighboring nodes. The normalization factor $\frac{1}{|N(v)|+1}$ ensures that the influence of each neighbor is appropriately scaled.

To ensure numerical stability and enable richer structural context aggregation, we stack two HGCN layers^26^, allowing each node to incorporate information from its two-hop neighborhood.

#### Deep-View Encoding (DV Encoder)

To capture hidden relationships embedded in complex multi-hop pathways—such as direct protein interactions and indirect connections mediated by drugs—within the heterogeneous network, we design a GWT module as the deep-view encoder.

**Fig. 5:**
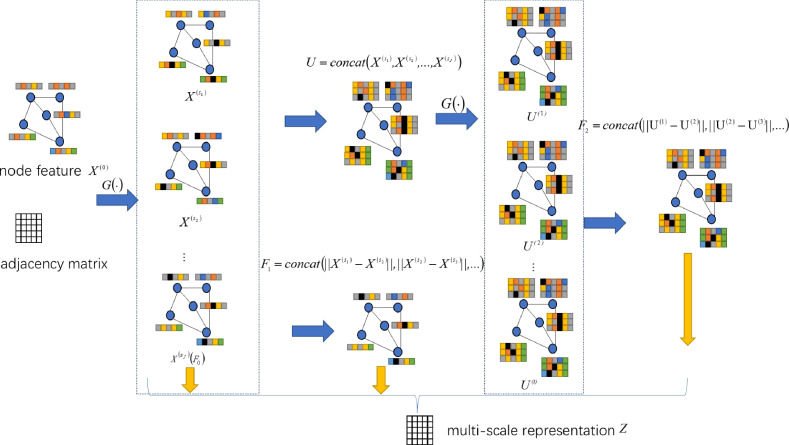
Visual illustration of the multi-scale graph wavelet transform, corresponding to the mathematical formulations in Eqs. (2)–(8). Given the input node features $X^{(0)}$ and the adjacency matrix, the model first performs multi-hop neighborhood aggregation using a propagation operator $G(\cdot )$ to obtain features at different scales $X^{(s_1)}, X^{(s_2)}, \ldots , X^{(s_J)}$. These features are concatenated to form a unified multi-scale representation $U$. To capture structural dynamics, the model computes first-order differences ($F_1$) between adjacent scale features and propagates $U$ further to derive higher-level features $U^{(1)}, U^{(2)}, \ldots , U^{(t)}$, from which second-order differences ($F_2$) are calculated. Finally, all features are concatenated and passed through a PReLU-activated linear layer to produce the final multi-scale representation $Z$. This approach enables the model to capture both local interactions and global graph structure.

In contrast to HGCN that perform direct neighborhood information aggregation, the proposed GWT module processes multi-hop relational paths via multi-scale graph signal decomposition.This approach facilitates the extraction of node representations across different frequency bands and hierarchical levels, capturing both local fine-grained interactions and global structural patterns.

We first perform multi-step weighted aggregation to derive node features at different scales. Let the operator $G(\cdot )$ denote a weighted neighborhood aggregation function. Given the node feature matrix $X \in {\mathbb {R}}^{N \times d}$, the propagation process is defined as:

2 $$\begin{aligned} X^{(t)} = G(X^{(t-1)}), \quad t = 1, 2, \ldots , T \end{aligned}$$

where $X^{(0)} = X$ is the initial input feature. For key scales $s_1, s_2, \ldots , s_J$, the corresponding multi-scale features are:

3 $$\begin{aligned} & U = \text {concat}(X^{(s_1)}, X^{(s_2)}, \ldots , X^{(s_J)}) \end{aligned}$$

4 $$\begin{aligned} & s_J = 2^{J-1} \end{aligned}$$

We set $J = 3$ in experiments, corresponding to 1-hop, 2-hop, and 4-hop propagations. Afterward, to model feature dynamics across scales, we compute both first-order and second-order differences. Let:

5 $$\begin{aligned} {\textbf{U}}^{(0)} = {\textbf{U}}, \quad {\textbf{U}}^{(t)} = G({\textbf{U}}^{(t-1)}), \quad t = 1, \ldots , M \end{aligned}$$

We define the first-order difference as:

6 $$\begin{aligned} {\textbf{F}}_1 = \text {concat} \left( \Vert X^{(s_1)} - X^{(s_2)} \Vert , \Vert X^{(s_2)} - X^{(s_3)} \Vert , \ldots \right) \end{aligned}$$

and the second-order difference as:

7 $$\begin{aligned} {\textbf{F}}_2 = \text {concat} \left( \Vert U^{(1)} - U^{(2)} \Vert , \Vert U^{(2)} - U^{(3)} \Vert , \ldots \right) \end{aligned}$$

The final multi-scale representation *Z* is obtained by concatenating features and applying a fully connected layer with PReLU activation:

8 $$\begin{gathered} Z = {\text{PReLU}}({\text{concat}}(X^{{(s_{3} )}} ,\mathop {\underbrace {{{\text{concat}}_{{i = 1}}^{2} \left| {X^{{(s_{i} )}} - X^{{(s_{{i + 1}} )}} } \right|}}_{{}}}\limits_{{F_{1} }} , \hfill \\ \quad \quad \quad \mathop {\underbrace {{{\text{concat}}_{{i = 1}}^{2} \left| {U^{{(i)}} - U^{{(i + 1)}} } \right|}}_{{}}}\limits_{{F_{2} }} )W + b) \hfill \\ \end{gathered}$$

Here, *W* and *b* are trainable parameters, and $|\cdot |$ denotes element-wise absolute difference.The above formulas, Eqs. (2)–(8), are visually illustrated in Fig. 5 for better understanding.

To enhance the semantic expressiveness of multi-scale features, we adopt a semantic attention mechanism. Each GWT-derived feature set is first projected through a non-linear transformation and then aggregated across nodes. Attention scores are computed via a learnable vector *a* and used to weight each view’s features:

9 $$\begin{aligned} \alpha _i = \frac{ \exp \left( a^{T} \left( \frac{1}{N} \sum _{j=1}^{N} \tanh (W z_{i,j} + b) \right) \right) }{\sum _{k=1}^{P} \exp \left( a^{T} \left( \frac{1}{N} \sum _{j=1}^{N} \tanh (W z_{k,j} + b) \right) \right) } \end{aligned}$$

10 $$\begin{aligned} Z_{\text {fused}} = \sum _{i=1}^{P} \alpha _i Z_i \end{aligned}$$

Here, $Z_i \in {\mathbb {R}}^{N \times d}$ denotes the representation matrix for the *i*-th view, and $Z_{\text {fused}}$ is the weighted fused representation.

By leveraging wavelet-based multi-scale processing, the DV Encoder enables layer-wise interpretability. Low-frequency components correspond to stable topological backbones, while high-frequency components highlight dynamic or discriminative signals, thereby offering interpretable insights into latent binding mechanisms in drug–target interactions.

#### Multi-level contrastive learning

Recent studies have demonstrated that contrastive learning frameworks—such as PDGCL-DTI, which integrates parallel global-local contrast, and CDPMF-DDA, which employs multi-view contrast—can effectively enhance drug-target or drug-disease predictions by leveraging structural and semantic diversity across biomedical networks^27,28^.

Multi-level contrastive learning, a self-supervised learning paradigm, aims to capture intrinsic data structures by minimizing the representation distance between augmented views of the same sample while maximizing the divergence between different samples. This approach significantly reduces reliance on large-scale labeled data and generates high-quality supervisory signals for complex network tasks.

In the context of drug–target interaction prediction, our model extracts complementary features through: (i) local neighborhood information via the NV Encoder module, and (ii) cross-type, multi-hop relational patterns via the DV Encoder module. By aligning these multi-perspective node representations through contrastive learning, we enhance feature complementarity, ultimately improving model robustness and generalization performance.

Within our contrastive learning framework, we propose an intelligent positive sample selection strategy based on multi-order semantic paths. The methodology involves three key components:

1. Construction of path-specific similarity matrices for diverse multi-hop relational patterns (e.g., *drug–drug*, *drug–protein–drug*), followed by normalization and identity matrix augmentation to preserve node self-similarity.

2. Integration of these matrices into a composite multi-order similarity matrix that comprehensively encodes heterogeneous network relationships.

3. Implementation of a dynamic thresholding mechanism where nodes exceeding type-specific cardinality thresholds (Top-10 for drugs, Top-5 for proteins) undergo similarity-based selection, while retaining all candidates below the threshold.

For negative sample selection, we primarily utilize implicit negatives generated within mini-batches, as they inherently provide diverse and moderately challenging contrastive signals. A detailed discussion and comprehensive comparison of both positive and negative sampling strategies can be found in the Appendix.

The final selections are systematically encoded in a binary indicator matrix $\texttt {pos\_dict}$, which provides robust alignment guidance for subsequent contrastive feature learning.

To formulate the contrastive loss, we unify the bidirectional contrastive objectives into a single formulation. For the *h*-th layer, the similarity between the DV and NV representations is defined as:

11 $$\begin{aligned} S_{ij}^{(h)} = \frac{ {\textbf{Z}}_{\text {DV},j}^{(h)} \cdot {\textbf{Z}}_{\text {NV},j}^{(h)} }{ \left\| {\textbf{Z}}_{\text {DV},j}^{(h)} \right\| \cdot \left\| {\textbf{Z}}_{\text {NV},j}^{(h)} \right\| \cdot \tau } \end{aligned}$$

Here, ${\textbf{Z}}_{\text {DV},j}^{(h)}$ and ${\textbf{Z}}_{\text {NV},j}^{(h)}$ are projection representations from the deep and neighborhood views, respectively. $\tau$ is the temperature coefficient, set to 0.5.

We use the positive sample indicator matrix *P* to define which node pairs are positives (i.e., $P_{ij} = 1$ if nodes *i* and *j* are positives). The contrastive loss for direction $\mu$ is given by:

12 $$\begin{aligned} L^{(h), \mu } = -\frac{1}{n} \sum _{k=1}^{n} \log \frac{ \sum _{l=1}^{n} \exp \left( S_{kl}^{(h), \mu } \right) P_{kl}^{(\mu )} }{ \sum _{l=1}^{n} \exp \left( S_{kl}^{(h), \mu } \right) } \end{aligned}$$

where $\mu \in \{ \text {DV} \rightarrow \text {NV}, \text {NV} \rightarrow \text {DV} \}$ indicates the alignment direction. Specifically, we define $S_{ij}^{(h), \text {DV} \rightarrow \text {NV}} = S_{ij}^{(h)}$ as the similarity from DV to NV, and analogously for the reverse direction.

The total multi-level contrastive learning objective is then expressed as:

13 $$\begin{aligned} L_{\text {contrast}} = \sum _{h=1}^{H} \frac{1}{2} \left[ L^{(h), \text {DV} \rightarrow \text {NV}} + L^{(h), \text {NV} \rightarrow \text {DV}} \right] \end{aligned}$$

This dual-view alignment strategy not only enhances the robustness of learned representations but also facilitates biological interpretability by encouraging consistency between topological proximity and functional similarity.

#### Drug–target prediction

Upon acquiring the feature representations of heterogeneous nodes (including drugs, proteins, diseases, and side effects), we employ the DistMult module to jointly model multiple relationships within the heterogeneous network, thereby learning more discriminative entity embeddings. The DistMult framework utilizes diagonal relationship matrices to simultaneously capture diverse entity interactions, enabling the learned embeddings to inherently encode multiple relational semantics through joint optimization. This process includes initializing relation-specific parameters, computing triplet scores for entity–relation–entity triples, and iteratively refining embeddings via gradient-based optimization.

**Diagonal matrices for relationship modeling.** DistMult defines a learnable diagonal matrix for each edge type. Since only diagonal elements are parameterized, the model maintains a low parameter count while allowing independent scaling of each embedding dimension. This structure enables the model to assign varying importance to different dimensions across relations. For any two types of nodes, their interaction is approximated via a bilinear operation. For example, the drug–protein interaction is modeled as:

14 $$\begin{aligned} G_{\text {re}}^{\text {dti}} = \Phi _{\text {drug}} \, \Theta _{\text {dti}} \, \Phi _{\text {protein}}^{T} \end{aligned}$$

Here, $\Phi _{\text {drug}}$ and $\Phi _{\text {protein}}$ represent the drug and protein embedding matrices, respectively. $\Theta _{\text {dti}}$ is a learnable diagonal matrix specific to the drug–target interaction. This modeling is equivalent to weighting each embedding dimension separately and computing their interactions, which allows the model to capture the contribution of different latent dimensions.

Similar formulations are applied to other types of relations in the heterogeneous network.

**Relation reconstruction and loss.** To supervise the training, each relation is reconstructed using the above formulation. The reconstruction quality is measured by the mean squared error (MSE) between the observed relationship matrix and its reconstructed counterpart. Specifically, for the drug–protein interaction:

15 $$\begin{aligned} L_{\text {dti}} = \left\| G^{\text {dti}} - G_{\text {re}}^{\text {dti}} \right\| ^2 \end{aligned}$$

For all relation types $r \in R$, the total reconstruction loss is aggregated as:

16 $$\begin{aligned} L_{\text {re}} = \sum _{r \in R} L_r \end{aligned}$$

**Final optimization objective.** Our overall model jointly optimizes three objectives: (i) reconstruction loss $L_{\text {re}}$ from DistMult, (ii) contrastive loss $L_{\text {cl}}$ from multi-level contrastive learning, and (iii) an $\ell _2$-norm regularization term $L_{\text {L2}}$. The final loss function is:

17 $$\begin{aligned} L = L_{\text {re}} + \lambda _1 L_{\text {cl}} + \lambda _2 L_{\text {L2}} \end{aligned}$$

Here, $\lambda _1 = 0.5$ and $\lambda _2 = 20{,}000$ are hyperparameters controlling the relative importance of each component.

This joint objective enables our model to not only learn robust and expressive multi-view node representations but also to reconstruct diverse relationships in the heterogeneous biomedical network, thereby improving drug–target interaction prediction performance.

### Experimental setup parameters

All experiments were conducted in a GPU-enabled environment. The model was trained for a maximum of 5,000 epochs, with early stopping applied if no improvement was observed over 500 consecutive validation rounds to mitigate overfitting. The node embedding dimension was set to 2,048, and the initial learning rate was 0.001. Dropout rates were set to 0.5 at the feature level and 0.2 at the attention level. For contrastive learning, the temperature parameter $\tau$ used in the cosine similarity computation was fixed at 0.5. The ratio of positive to negative samples in the drug–target interaction dataset was maintained at 1:10.

The proposed model outputs a drug–target interaction probability matrix defined over a heterogeneous biomedical graph, where each matrix entry quantifies the likelihood of interaction between a specific drug and protein. The original dataset consists of interaction triplets–each comprising a drug index, a protein index, and a binary interaction label.

To ensure a balanced evaluation, we employed a stratified 10-fold cross-validation strategy. In each fold, one partition served as the test set, while the remaining nine formed the training set. The training set was further split into 90% for model training and 10% for validation.

Model performance was primarily assessed using two metrics: the area under the receiver operating characteristic curve (AUC) and the area under the precision–recall curve (AUPR). AUC reflects the model’s ability to distinguish between positive and negative interactions, while AUPR emphasizes precision and recall trade-offs, offering a more informative evaluation in the presence of class imbalance.

## Experimental results

### Comparison with baseline models

We compare the performance of GHCDTI with several state-of-the-art DTI prediction models. These baselines represent diverse architectural paradigms, including hypergraph modeling, Siamese networks, convolutional encoders, and graph attention mechanisms:

- **FRoGS**^29^: Integrates gene ontology annotations and RNA-seq profiles to construct functional gene embeddings via hypergraph-based contrastive learning. A twin-network aligns drug-induced transcriptomic responses for interaction prediction.
- **SiamDTI**^30^: Based on a Siamese network that jointly encodes drugs and protein targets, with cross-domain feature fusion capturing both local and global structural cues.
- **HyperAttentionDTI**^31^: Utilizes stacked 1D convolutional layers and a multi-dimensional attention mechanism to encode drugs and proteins while improving interpretability.
- **DTI-GAT**^32^: Applies graph attention networks (GATs) on a heterogeneous DTI graph to learn topological dependencies and latent features for accurate predictions.
- **iGRLDTI**^33^: Proposes a node-dependent local smoothing (NDLS) strategy on a heterogeneous biological information network to adaptively determine propagation depth and alleviate over-smoothing in GNNs.

**Fig. 6:**
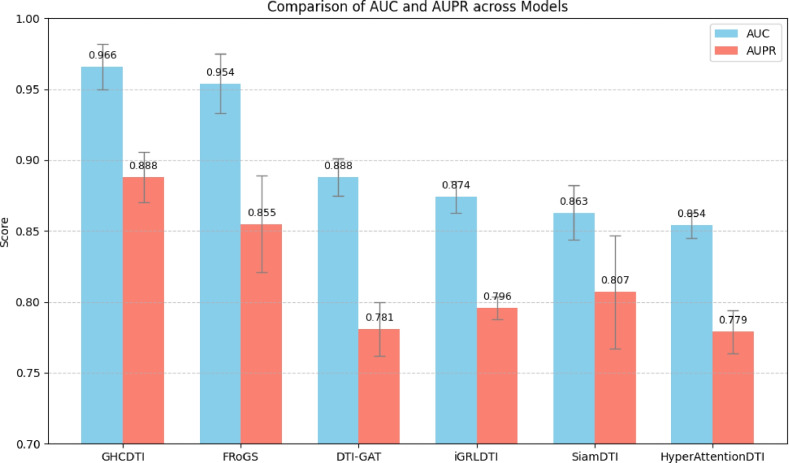
Performance comparison of different models on the Luo dataset.

As shown in Fig. 6, GHCDTI outperforms all baseline methods on AUC and AUPR metrics, demonstrating superior ability to identify true drug-target interactions. This advantage stems from its graph hypernetwork architecture, which explicitly models higher-order relations via hyperedges while preserving node identity through self-loops, enhancing representation expressiveness and alleviating oversmoothing. Additionally, GHCDTI integrates multi-scale structural features through graph wavelet transforms and heterogeneous data fusion, coupled with cross-view contrastive learning, enabling richer and more discriminative embeddings. These design choices enable GHCDTI to more effectively model complex biological relationships and dynamic binding patterns, demonstrating enhanced robustness and more consistent prediction performance compared to methods limited to pairwise interaction analysis.

### Bootstrap-based robustness evaluation

**Fig. 7:**
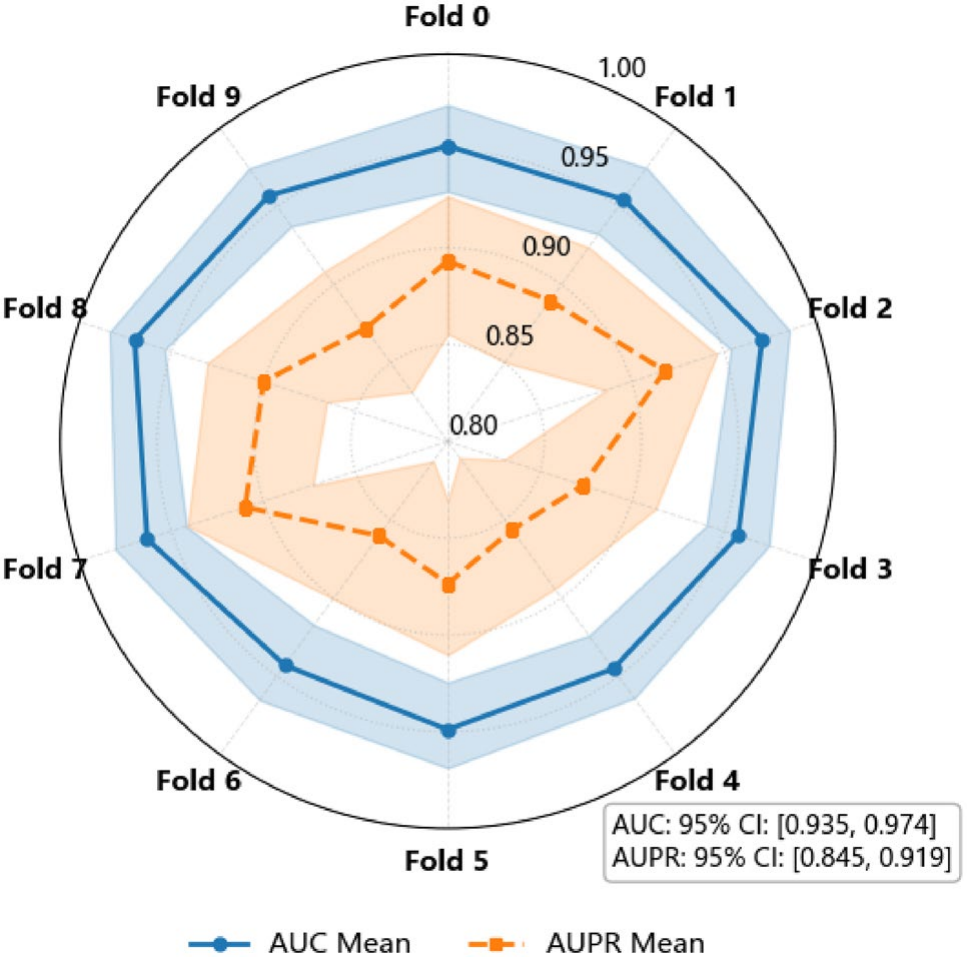
Polar plots showing AUC and AUPR across 10 folds. Blue lines and shaded areas represent AUC mean and 95% CI; orange lines and areas show AUPR mean and 95% CI. Each axis corresponds to one fold.

To evaluate robustness and generalization under varying data partitions, we performed 10-fold cross-validation and estimated 95% confidence intervals (CIs) for both AUC and AUPR using bootstrap resampling. The results are visualized in Fig. 7.

Bootstrap-based CIs offer a robust, non-parametric means to quantify predictive uncertainty, particularly valuable in biomedical domains with small and skewed datasets. As seen in Fig. 7, the AUC curves are highly stable with narrow confidence bands, indicating consistent classification capability across folds. In contrast, the AUPR curves exhibit slightly wider intervals, reflecting their sensitivity to class imbalance and variations in precision–recall trade-offs.

The consistent AUC and relatively stable AUPR values across folds suggest that GHCDTI is both robust and reliable, making it a promising candidate for real-world biomedical applications.

### Attention mechanism and interpretability

To investigate the role and interpretability of attention mechanisms in GHCDTI, we perform both quantitative and qualitative analyses.

First, we compare the proposed semantic attention module with four commonly used alternatives: similarity-based attention^34^, gated attention^35^, multi-head attention^36^, and scaled dot-product attention^37^. All other components and hyperparameters remain fixed throughout the experiments.

**Table 3:** Performance comparison of different attention mechanisms on the drug–target prediction task.

Attention Mechanism	AUC	AUPR
Semantic Attention	**0.966** ± **0.016**	**0.888** ± **0.018**
Similarity-Based Attention	0.955 ± 0.013	0.883 ± 0.006
Gated Attention	0.958 ± 0.017	0.884 ± 0.015
Multi-Head Attention	0.958 ± 0.009	0.883 ± 0.004
Scaled Dot-Product Attention	0.959 ± 0.015	0.885 ± 0.012

As shown in Table 3, all attention variants achieve competitive performance, highlighting the general value of attention-based modeling. However, semantic attention achieves the best AUC and AUPR, demonstrating the effectiveness of explicitly aligning multi-view semantics in heterogeneous biomedical graphs.

**Fig. 8:**
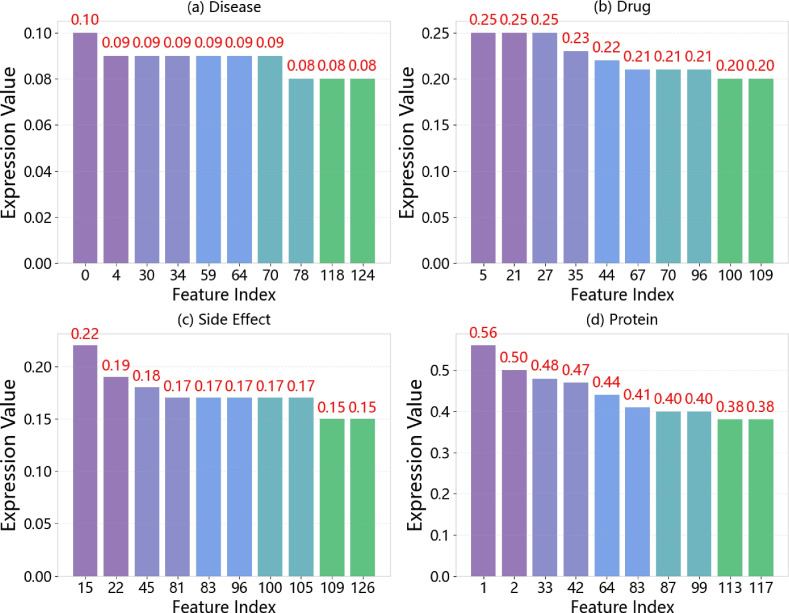
Distribution of attention weights across semantic views for different node types. The x-axis shows the 128 dimensions (semantic views), and the y-axis shows average attention scores.

To further interpret the behavior of semantic attention, we visualize the average attention weights assigned to different semantic views across four types of nodes. Figure 8 shows the distribution of attention scores across 128 semantic dimensions over the course of training.

Protein embeddings exhibit strong selectivity, with the first dimension receiving the highest attention score (0.56), followed by a gradual decay. This suggests the model captures essential structural motifs. In contrast, drug embeddings show a more distributed pattern with notable peaks (e.g., dimensions 5, 21, 27), reflecting diverse chemical substructures.

Side effect and disease nodes present flatter distributions, with maximum weights of 0.19 and 0.09, respectively. These patterns imply that while proteins and drugs carry primary predictive signals, side effects and diseases play an auxiliary role by providing contextual information.

Overall, these findings highlight the interpretability of GHCDTI, showcasing how the model differentially allocates attention in accordance with biological relevance across heterogeneous node types.

### Hyperparameter sensitivity analysis

We conduct a comprehensive study on three critical hyperparameters in GHCDTI: the hidden embedding dimension *d*, the $L_2$ regularization weight $\lambda _1$, and the contrastive loss weight $\lambda _2$ (denoted as cl).

**Fig. 9:**
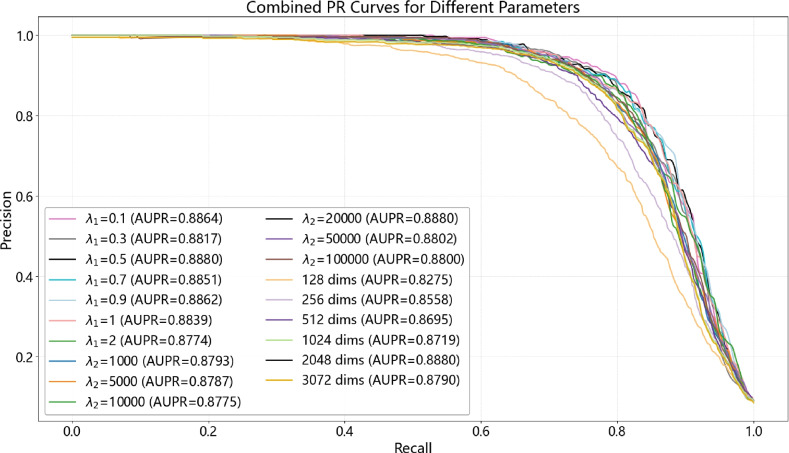
Precision–Recall (PR) curves of GHCDTI under different hyperparameter settings: embedding dimension *d*, regularization coefficient $\lambda _1$, and contrastive loss weight $\lambda _2$.

Figure 9 presents the combined PR curves demonstrating the impact of varying these key hyperparameters on model performance.

#### Embedding dimension *d*

We evaluate $d \in \{128, 512, 1024, 2048, 3072\}$. Increasing *d* consistently improves AUPR, reaching a peak of 0.888 at $d=2048$. Further increasing to $d=3072$ offers only marginal gains while substantially increasing computational cost, indicating $d=2048$ is the optimal trade-off between accuracy and efficiency.

#### Regularization coefficient $\lambda _1$

We evaluated the impact of the regularization coefficient $\lambda _1$ using a range of values: $\lambda _1 \in \{0.1, 0.3, 0.5, 0.7, 0.9, 1, 2\}$. Performance improved steadily as $\lambda _1$ increased up to 0.5, indicating effective prevention of overfitting. However, further increases led to a decline in performance, suggesting that excessive regularization constrained the model’s capacity. The best performance was observed at $\lambda _1 = 0.5$, striking a balance between model expressiveness and regularization strength.

#### Contrastive loss weight $\lambda _2$

Varying $\lambda _2$ across $\{1\,000, 5\,000, 10\,000, 20\,000, 50\,000, 100\,000\}$ shows gradual performance improvement up to $\lambda _2 = 20\,000$, where the AUPR is optimally adjusted to 0.888. Further increases result in diminishing or negative returns, likely due to excessive contrastive loss dominating the training signal and hindering drug–target interaction prediction.

#### Positive sample Top-*K* selection

**Fig. 10:**
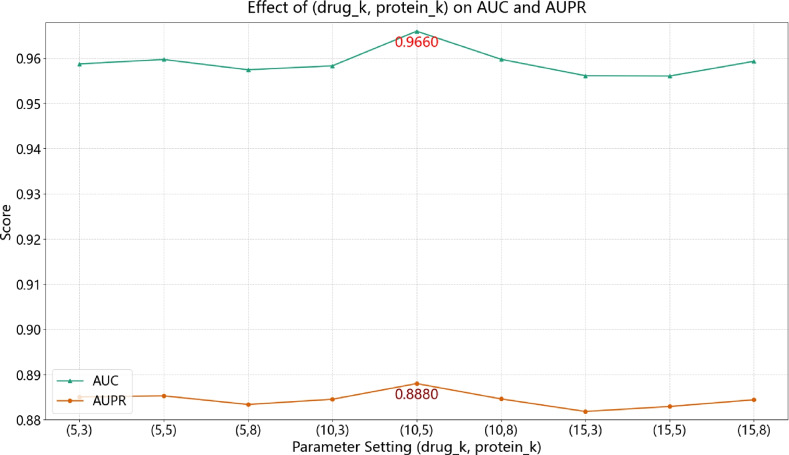
PR curves of GHCDTI under different top-*K* settings for contrastive positive sample selection.

In our contrastive learning module, we explored a wide range of values for the number of top-*K* most similar nodes used as positive samples, aiming to encourage semantically meaningful alignment. After evaluating various combinations of *K* for drug and protein nodes, we selected the best-performing settings for visualization and analysis. Specifically, we varied $K \in \{5, 10, 15\}$ for drugs and $K \in \{3, 5, 8\}$ for proteins, and present the top-performing configurations in Fig. 10.

The performance improves with increasing *K* up to a moderate value (e.g., $K=10$ for drugs and $K=5$ for proteins), beyond which it either saturates or slightly declines. This suggests that while a small *K* may insufficiently capture neighborhood semantics, an overly large *K* may introduce semantically weak or noisy neighbors, diluting the contrastive signal.

We restrict the analysis to drugs and proteins for two reasons: (1) they are the primary node types involved in drug-target interaction (DTI) prediction, and (2) disease and side effect nodes exhibit higher sparsity and weaker pairwise semantics, making their top-*K* selection less reliable. Additionally, preliminary experiments show that including contrastive objectives for these auxiliary nodes does not improve–and may even degrade–overall performance.

Thus, we set $K=10$ for drugs and $K=5$ for proteins as default values, which achieve a favorable trade-off between expressiveness and robustness in contrastive representation learning.

##### Summary

These findings highlight the importance of hyperparameter tuning in maximizing GHCDTI’s effectiveness. Specifically, the configuration $(d, \lambda _1, \lambda _2) = (2048, 0.5, 20000)$ consistently yields optimal results and is adopted as the default setting in all subsequent experiments.

### Cold start evaluation on unseen drugs and targets

To rigorously evaluate the generalization capability of GHCDTI in realistic biomedical settings, we conduct cold start experiments—a protocol widely used in recommender systems and increasingly applied to DTI prediction^38–40^. In this setup, the model is evaluated on entirely unseen drugs or protein targets, mimicking the challenge of predicting interactions for novel compounds or uncharacterized proteins.

We consider two distinct cold start scenarios:

- **Cold Drug**: All drugs in the test fold are completely excluded from both training and validation sets.
- **Cold Target**: All protein targets in the test fold are unseen during training and validation.

Following prior work^40,41^, we adopt a 10-fold entity-level cross-validation protocol. Specifically, the 708 drugs (or 1512 targets) are randomly partitioned into 10 disjoint folds. In each iteration, one fold is used for testing, and the remaining folds are split (90%:10%) into training and validation. We strictly ensure that no test entity is included in any structural or multi-hop relational patterns derived during training.

Negative samples are generated at a 10:1 ratio to positives, with high-similarity false negatives excluded. Graph topology, multi-hop relational information, and embeddings are constructed solely from training data in each fold. Each experiment is conducted with three random seeds, and results are reported as the average performance.

**Table 4:** Performance comparison of GHCDTI under different cold start evaluation settings. The best results are highlighted in bold.

Evaluation Setting	AUC	AUPR
**Normal**	**0.966** ± **0.016**	**0.888** ± **0.018**
**Cold Drug**	0.920 ± 0.024	0.778 ± 0.065
**Cold Target**	0.881 ± 0.037	0.691 ± 0.088

As presented in Table 4, GHCDTI demonstrates strong performance under the standard evaluation setting. In the cold drug scenario, the model experiences a moderate decline–AUC drops by 4.8% and AUPR by 12.4%–indicating a degree of generalization enabled by molecular structure, albeit constrained by the lack of historical interaction data.

In contrast, the cold target setting results in more substantial performance degradation (AUC: –8.8%, AUPR: –22.2%), highlighting the greater challenge in extrapolating to entirely unseen proteins. This finding aligns with previous studies^40^ and underscores the limitations of existing representation methods when faced with novel biological entities. These results point to promising directions for future work, such as leveraging pre-trained protein language models (e.g., ESM, ProtBERT) or structure-based embeddings from AlphaFold to enhance target generalization.

Overall, our cold-start protocol offers a reproducible and rigorous benchmark for assessing model robustness in realistic DTI prediction scenarios.

### Ablation study

To comprehensively assess the individual contributions of each component within the GHCDTI framework, we systematically designed a series of ablation studies. The experimental outcomes are quantitatively summarized in Table 5 and Table 6, with key findings detailed below:

**Full Model (GHCDTI)**

- Configuration: Heterogeneous Graph Convolutional Network (HGCN) + Graph Wavelet Transform (GWT) + Multi-level Contrastive Learning.
- Purpose: Serves as the baseline, integrating all proposed innovations.

**GWT Ablation (HGCN + Contrastive Learning)**

- Modification: GWT module replaced with a standard Graph Convolutional Network (GCN).
- Rationale: Isolates the impact of multi-scale frequency-domain feature extraction.

**Contrastive Learning Ablation**

- Modification: Removed the contrastive learning module (HGCN + GWT only).
- Rationale: Isolates the effect of cross-view representation alignment on generalization.

**Single-Layer HGCN Variant**

- Modification: Replaced two-layer HGCN with a single-layer architecture.
- Hypothesis: Tests whether multi-hop neighborhood aggregation is essential for capturing heterogeneous relations.

**Single-Scale GWT Variant**

- Modification: Restricted GWT to a single wavelet scale.
- Objective: Evaluates the necessity of multi-scale frequency decomposition.

**Reduced-Scale GWT (J=2)**

- Modification: Limited GWT to two wavelet scales .
- Focus: Quantifies the trade-off between computational cost and multi-scale feature extraction.

**Table 5:** Ablation study results on the Luo Dataset (Mean ± Std).

Model	AUC	AUPR
Single-layer GWT	0.959 ± 0.012	0.885 ± 0.014
Single-layer HGCN	0.956 ± 0.007	0.880 ± 0.003
No Contrast	0.958 ± 0.004	0.878 ± 0.006
GWT (J=2)	0.953 ± 0.007	0.867 ± 0.007
No GWT (use GCN)	0.948 ± 0.010	0.859 ± 0.006
**GHCDTI**	**0.966** ± **0.016**	**0.888** ± **0.018**

**Table 6:** Ablation study results on the Zeng Dataset (Mean ± Std).

Model	AUC	AUPR
Single-layer GWT	0.961 ± 0.009	0.886 ± 0.006
Single-layer HGCN	0.962 ± 0.002	0.868 ± 0.011
No Contrast	0.969 ± 0.003	0.898 ± 0.001
GWT (J=2)	0.965 ± 0.004	0.888 ± 0.002
No GWT (use GCN)	0.965 ± 0.017	0.898 ± 0.016
**GHCDTI**	**0.977** ± **0.009**	**0.901** ± **0.007**

On the Luo dataset, the full model achieves best results (AUC = 0.966, AUPR = 0.888). Removing GWT results in a 1.77% drop in AUC and 4.40% in AUPR, confirming the importance of multi-scale wavelet features. Excluding contrastive learning causes smaller drops (AUC: –1.04%, AUPR: –0.90%), indicating its complementary benefit. Using a single-layer HGCN reduces AUC by 1.04%, while a single-layer GWT leads to minor performance loss.

On the Zeng dataset, GHCDTI achieves AUC = 0.977 and AUPR = 0.901. Ablating GWT or contrastive learning yields smaller degradations, suggesting that this dataset benefits less from these modules. However, single-layer HGCN leads to a larger performance drop (AUC: –1.50%, AUPR: –2.80%), highlighting the necessity of deeper neighborhood aggregation in more complex settings.

These results confirm that both GWT and contrastive learning are critical for performance, especially in structurally diverse datasets. GWT captures multi-scale semantic topology, while contrastive learning reinforces representation alignment. Multi-layer HGCN consistently outperforms shallower versions by aggregating richer context from heterogeneous graphs.

## Discussion

### Biological significance

The GHCDTI model not only outperforms various state-of-the-art DTI prediction models in terms of performance metrics but also demonstrates strong biological interpretability. The visualization of attention weight distributions reveals that for protein features, high weights are concentrated on specific structural regions, indicating the model’s ability to identify critical residues associated with actual binding sites. In contrast, drug features exhibit a more balanced weight distribution, suggesting that the model effectively integrates multiple physicochemical properties of compounds. Side effect and disease nodes receive relatively lower attention weights, reflecting their auxiliary roles in the prediction task. Through the incorporation of semantic attention mechanisms for multi-source feature fusion, the model captures both conserved structural backbones (low-frequency components) and functional variability (high-frequency components), providing mechanistic insights into drug-target interactions. Furthermore, the model demonstrates high computational efficiency, completing the processing of over 1,500 proteins and 700 drugs within a short time span, making it a practical and scalable tool for large-scale virtual screening, target identification, and drug repurposing applications.

### Limitations

Despite its superior performance, GHCDTI still presents several limitations. First, the current model is built upon a static heterogeneous network and does not incorporate the dynamic conformational changes of proteins or the pharmacokinetic processes of drugs, limiting its capacity to model transient or time-dependent interactions. Second, the initial features of drugs and proteins are primarily derived from SMILES strings and amino acid compositions, which may not fully capture three-dimensional conformational nuances or the influence of cellular environments, potentially hindering generalization to novel chemical entities. Third, although the semantic attention mechanism enhances interpretability, it relies on abstract feature dimensions without direct biological annotations, and thus still requires validation through experimental biology. Additionally, while contrastive learning improves feature alignment across multiple views, its robustness may be challenged under conditions of extreme class imbalance or the presence of out-of-distribution samples.

### Future directions and challenges

While GHCDTI demonstrates strong performance and interpretability, several avenues remain for future enhancement:

Dynamic interaction modeling: The current static graph representation could be enhanced with temporal graph networks (TGNs) or dynamic attention mechanisms to better capture the temporal evolution of drug-target binding processes. Related works, such as NCH-DDA^42^, have shown that integrating neighborhood-level structural variations with contrastive learning can improve representation robustness in sparse or evolving biomedical networks, suggesting potential benefits for dynamic DTI modeling as well.

3D structural integration with geometric priors: Although GHCDTI utilizes protein structure graphs, it does not explicitly enforce geometric invariance. Future work could integrate graph wavelet transforms with SE(3)-equivariant geometric deep learning models, which are inherently sensitive to the spatial symmetries of molecular structures. By leveraging high-resolution protein structures predicted by AlphaFold3 and similar tools, such integration could substantially improve the precision of binding site prediction and the model’s robustness to orientation and conformation variability. In this context, KSGTN-DDI^43^ demonstrates the value of explicitly modeling key substructure importance using adaptive graph Transformers, suggesting that combining substructure-aware encoding with spatially equivariant representations could further enhance DTI prediction. This approach may also facilitate the discovery of cryptic pockets and allosteric sites often overlooked by purely topological methods.

Privacy-preserving frameworks: The contrastive learning approach could be extended to federated learning para-digms to enable secure, distributed training while protecting sensitive drug data. In addition, future directions may explore robust contrastive representation learning under label noise or low-quality samples, which is common in biomedical settings. For example, M3C^44^ demonstrates that selecting core samples based on mutual information and integrating inter-category contrastive learning can significantly improve predictive robustness even with limited, noisy data. Similar strategies could be adapted in GHCDTI to enhance generalization in low-resource or semi-supervised drug discovery scenarios.

Overall, these directions open up exciting possibilities for extending GHCDTI into more dynamic, structure-aware, and privacy-compliant scenarios in real-world drug discovery pipelines.

## Conclusion

In this study, we propose GHCDTI, a novel framework that integrates graph wavelet transform and heterogeneous contrastive learning for accurate DTI prediction. GHCDTI leverages a *NV Encoder* to model edge-type-specific semantic relations via independent HGCN layers, and a *DV Encoder* to extract multi-scale structural features through a wavelet-based encoder with contrastive objectives. These two complementary views are jointly optimized, enabling the model to capture both semantic and structural contexts in heterogeneous biomedical graphs.

Extensive experiments on multiple benchmark datasets demonstrate that GHCDTI significantly outperforms state-of-the-art baselines under both general and cold-start scenarios. Furthermore, the proposed semantic attention mechanism and modular design enhance the model’s interpretability, allowing visualization of attention weights and disentangled representation paths. Ablation studies confirm the effectiveness of each component, and the interpretability analysis shows GHCDTI’s capacity to provide biologically meaningful insights.

Overall, GHCDTI represents a promising and interpretable approach to modeling DTI, with potential applications in drug repurposing and biomedical knowledge graph mining.

## Supplementary Information

Supplementary Information.

## Data Availability

Data is provided within the manuscript . All data used in this study are publicly available. One dataset was originally published by Luo et al. in Nature Communications, which integrates drug-target interactions,drug-disease associations, and protein-protein interactions from multiple public sources. It is available at: https://github.com/luoyunan/DTINet. Another dataset was published by Zeng et al. in Chemical Science, which contains curated drug-target interaction data derived from heterogeneous biomedical networks. It is available at: https://github.com/ChengF-Lab/deepDTnet.
