# Supplementary material for: Heterogeneous network drug-target interaction prediction model based on graph wavelet transform and multi-level contrastive learning
Source: Sci Rep. 2025 Aug 19;15:30326. doi: 10.1038/s41598-025-16098-y (PMC12365291; doi:10.1038/s41598-025-16098-y)
Supplement: Supplementary file 1 — Supplementary Information. [file 41598_2025_16098_MOESM1_ESM.pdf]

Appendix

1 Comparison and Analysis of Positive and Negative Sampling Strategies

In heterogeneous graph contrastive learning, the construction of positive and negative sample pairs plays a critical role in determining the quality of learned representations. Appropriate positive sampling facilitates semantic aggregation, while well-designed negative samples enhance discriminability. Conversely, improper sampling may cause representation collapse, hinder convergence, or impair generalization. Therefore, the design of effective sampling strategies is crucial for improving model performance.

In this study, we systematically evaluate various positive and negative sampling strategies along two dimensions:

Positive Sampling Strategies

- **TOP-*k* Similarity Sampling**: Selects the top-*k* most similar intra-class nodes based on a single-view similarity matrix.
- **PCA-Fused Sampling**: Applies PCA to similarity matrices derived from multiple multi-hop pathways to extract a unified semantic representation, then selects top-*k* neighbors.
- **Diffusion-Augmented Sampling**: Enhances similarity via multi-step graph diffusion to capture indirect structural proximity, followed by top-*k* selection.

Negative Sampling Strategies

- **Random Sampling**: Randomly selects negatives excluding those in the positive set.
- **Hard Sampling**: Selects node pairs with zero similarity across all meta-paths, maximizing dissimilarity.
- **Semi-Hard Sampling**: Chooses nodes with moderate similarity, typically in the 25th-50th percentile range, balancing informativeness and difficulty.
- **Low-Similarity Sampling**: Filters negatives with low similarity based on multi-view aggregation.
- **No Explicit Negatives (None)**: Does not construct explicit negative pairs. Instead, it relies on in-batch sampling where other samples in the same mini-batch are treated as negatives, allowing the model to focus on maximizing the similarity of positive pairs while implicitly maintaining contrast.

Figure 1 presents the AUC and AUPR performance across all combinations of positive and negative sampling strategies. Notably, the combination of TOP-*k* positive sampling with no explicit negatives achieves the best results (AUC: 0.9660, AUPR: 0.8880), underscoring that well-chosen semantic positives alone are sufficient to drive effective contrastive learning, even in the absence of manually constructed negatives.

The TOP-*k* positive strategy selects semantically closest neighbors based on multi-view meta-path similarity, promoting intra-class compactness and preserving heterogeneous structural semantics. The absence of explicit negatives allows the model to rely on implicit in-batch negatives, which offer naturally diverse and moderately challenging contrastive signals. This design avoids two major pitfalls: (1) trivial negatives from random sampling, which offer weak learning signals; and (2) overly hard negatives, which often destabilize optimization and degrade performance.

In summary, positive sampling should prioritize semantic specificity and structural consistency to ensure meaningful intra-class alignment, while negative sampling should avoid both trivial (random) and overly hard examples to preserve training stability and contrastive informativeness.

These findings offer empirical guidance for constructing high-quality contrastive pairs in heterogeneous biomedical graphs, ultimately enhancing the effectiveness of contrastive learning in complex biological systems.

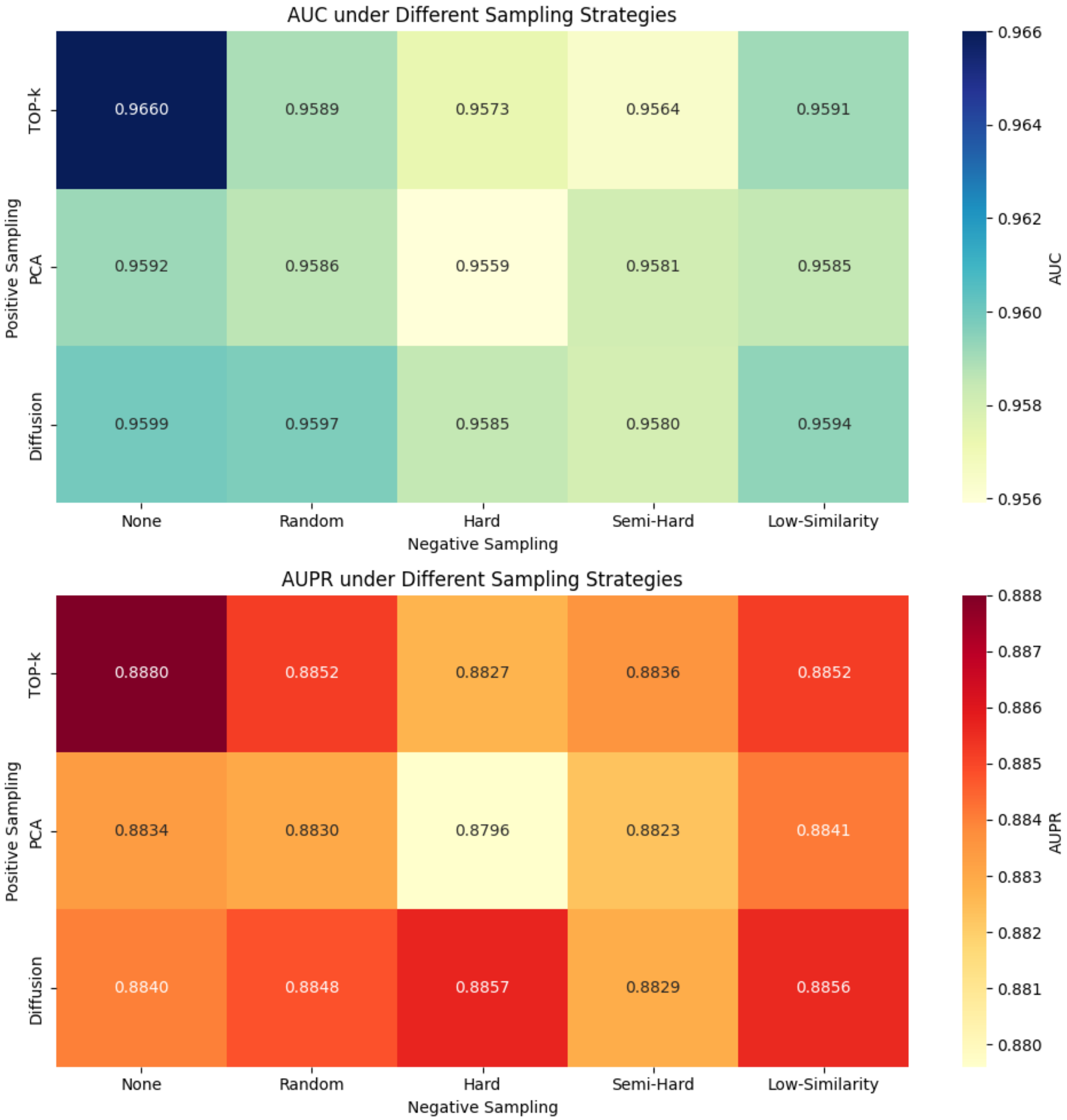

Figure 1. Comparison of AUC and AUPR under different sampling strategies. The best scores are highlighted in each submatrix.
